# Effect of longevity genotype on acute phase response to a lipopolysaccharide challenge in dairy goats

**DOI:** 10.1371/journal.pone.0356779

**Published:** 2026-08-28

**Authors:** Marie Ithurbide, Rachel Rupp, Thierry Fassier, Nicolas C. Friggens, Gilles Foucras

**Affiliations:** 1 GenPhySE, Université de Toulouse, INRAE, Castanet-Tolosan, France; 2 GABI, INRAE, AgroParisTech, Université Paris-Saclay, Jouy-en-Josas, France; 3 Domaine de Bourges, INRAE, Osmoy, France; 4 PEGASE, INRAE, Institut Agro, Saint-Gilles, France; 5 MoSAR, INRAE, AgroParisTech, Université Paris-Saclay, Gif-sur-Yvette, France; 6 Univ Toulouse, ENVT, INRAE, IHAP, Toulouse, France; University of Illinois, UNITED STATES OF AMERICA

## Abstract

The present study aims to explore the phenotypic variability of the inflammatory response to a lipopolysaccharide (LPS) challenge in goats and compare the reaction between two lines divergently selected for functional longevity. Hyper-selection based on functional longevity successfully created two groups of goats with different lifespans (High_LGV and Low_LGV). Primiparous goats were injected intravenously with LPS to trigger an inflammatory response, with a total of 83 goats, including 42 High_LGV and 41 Low_LGV goats. Blood was sampled 12 times at −72, 0 (injection), 2, 4, 6, 8, 10, 12, 24, 48, 72, and 144 hours. Baseline cytokine concentrations (average of hours −72 and 0) showed no significant differences between longevity lines for any of the 14 measured cytokines. Among the six cytokines that responded to the LPS challenge (IFN-γ, IL4, IL-6, IL-10, MIP1α, and TNFα), three showed significant differences between longevity lines in their temporal response profiles. Low_LGV goats exhibited stronger acute inflammatory responses with greater IL-6 and IFN-γ concentrations during the early hours post-challenge, while High_LGV goats showed elevated MIP1α concentrations during the resolution phase. In parallel, whole blood was stimulated for 24 hours with LPS on a total of 157 goats for ex vivo assessment of the blood response. The IL-6 concentration measured in the whole blood assay showed a moderate correlation with the in vivo response and revealed no difference between the longevity lines. These results suggest that a stronger acute inflammatory response, characterised by elevated pro-inflammatory cytokine production, may be detrimental to functional longevity in dairy goats, possibly through increased tissue damage and impaired recovery following inflammatory challenges.

## Introduction

Environmental perturbations, including pathogen exposure, are likely to increase in the future, and there is a growing interest in selecting more resilient livestock. Literature suggests that immunity is a key component of the underlying mechanisms of resilience. Indeed, infectious diseases remain a major cause of animal death and production losses, alongside metabolic and nutritional disorders [1]. In dairy ruminants, positive genetic correlations between immune responsiveness and health in cattle and sheep have been reported [2,3]. The genetic determinism of resistance to mastitis has been established [4–6], and its association with longevity has been highlighted in several studies. For example, Sasaki [7] presented significant genetic correlations between milk somatic cell score (SCS) and functional longevity. Functional longevity is defined as the lifespan of an individual animal adjusted for production level [7]. It is not only a desirable outcome of resilience but also stands as the foremost indicator of resilience [8]. It reflects an animal’s capacity to sustain productivity and well-being over its productive lifetime, offering valuable insights into resilience. Animals exhibiting prolonged functional longevity are more likely to possess inherent resilience mechanisms, enabling them to endure and adapt to environmental disruptions. Ithurbide et al. [9] found that a line of dairy goats hyperselected for high functional longevity had significantly lower SCS than a line selected for low functional longevity, highlighting the link between mammary inflammation and survival. The same study showed that a high concentration of blood immunoglobulins during the first week of life, reflecting passive immunity transfer through colostrum intake, was significantly associated with better survival during the first years of life of the dairy goats. In rabbits, breeding lines selected for reproductive longevity exhibit greater immune modulation and improved responses to immune challenges compared to lines selected exclusively for litter size [10,11], suggesting that selection criteria prioritizing longevity over productivity can enhance immunological robustness and disease resilience. Currently, there is limited understanding of how dairy goats’ immune responses vary when confronted with the same disease or inflammatory challenge, how these responses influence overall health, mammary condition, and longevity, and what genetic factors govern these immune reactions.

Cytokines are immunomodulatory polypeptides that play crucial roles in both inflammatory and immune responses. These signaling molecules facilitate communication between immune cells and coordinate complex defence mechanisms. Investigating cytokine dynamics is fundamental to elucidating immune function and associated sophisticated responses to pathogens, particularly those eliciting inflammatory reactions [12].

The experiment is based on two divergent lines of goats for extreme functional longevity (High_LGV and Low_LGV) that were previously characterised [9]. We hypothesise that the two Alpine goat lines exhibit distinct immune and inflammatory response profiles and that these differences represent a key component of the hereditary mechanisms explaining the superior survival observed in the High_LGV line. To test this hypothesis, a controlled experiment using an intravenous lipopolysaccharide (LPS) challenge as a model of an inflammatory reaction was conducted to examine the acute phase response. LPS challenge is a good model for studying the acute phase response in several species [13–15]. It induces clinical signs related to endotoxemia, such as fever, general depression, and marked changes in the expression of several inflammatory mediators. This standardised inflammatory stimulation allows for precise measurement of temporal cytokine dynamics and other immune parameters, enabling direct comparison between animals with genetically divergent longevity traits. Moreover, a whole blood assay with LPS may constitute a non-invasive alternative to animal challenge and has been implemented at the same time for comparison. This study aims to explore the link between immune traits related to the inflammatory response and dairy goat functional longevity.

## Materials and methods

### Animal management

The experiment was carried out in agreement with French National Regulations for the humane care and use of animals for research purposes. This article followed the ARRIVE guidelines 2.0 [16]. All procedures performed on animals were approved by the Ethics Committee on Animal Experimentation and the French Ministry of Higher Education, Research and Innovation (APAFIS#23183–2019120512104160 v13). Humane endpoints were predefined in the approved protocol. On the day of LPS injection, animal health was monitored every 2 hours from T0 to T12, then once daily until day 15. A clinical scoring system was applied: a score of 1 was attributed for each of the following signs: rectal temperature exceeding 41.5°C for more than 2 consecutive hours, body weight loss greater than 10%, or lateral decubitus. Animals with a cumulative score of≥2 were removed from the experiment and treated. If the animal failed to recover, euthanasia would be performed. All personnel involved in experimental procedures held valid certification in laboratory animal science and were up to date with continuing education requirements, in accordance with French regulations.

Following the method developed by [17] and described by [9], two functional longevity lines of Alpine goats have been created at INRAE. Since 2017, a genetic evaluation for functional longevity has been conducted on 8,787 alpine artificial insemination (AI) bucks based on the productive longevity of their daughters (time difference between first kidding and culling) corrected for milk yield. The 16 bucks that had the highest estimated breeding value (EBV) and the 19 bucks that had the lowest EBV were selected among the entire AI buck population to found the low longevity line (Low_LGV) and high longevity line (High_LGV), respectively.

The present experiment was conducted across three INRAE experimental facilities. At facility 1 (La Sapinière, Osmoy, France) and Facility 2 (P3R, Bourges, France https://doi.org/10.15454/1.5483259352597417E12, C18–174–01) housed goats that underwent both the in vivo and ex vivo challenges. Facility 3 (Grignon, Thiverval-Grignon, France, A 78 615 1002) housed goats that underwent only the ex vivo challenge. From 2020 to 2023, a total of 157 goats were bred across these three facilities: 73 Low_LGV and 84 High_LGV goats (Table 1).

**Table 1:** Distribution of the 157 goats within the 2 divergent lines selected on high longevity (High_LGV) or low longevity (Low_LGV) bred at INRAE facilities that underwent ex-vivo inflammatory challenge.

		Farm 1	Farm 2	Farm 3 Year 1	Farm 3 Year 2	*Total*
**Longevity line**	**Low_LGV**	18	24	17	*15*	*74*
	**High_LGV**	27	17	17	*22*	*83*
	***Total***	*45*	*41*	34	*37*	*157*

General facility management was similar in the three facilities and was described in [9]. Briefly, in each facility, Low_LGV and High_LGV goats were housed in common pens with no pasture access. Reproduction was seasonal, with goats inseminated at approximately 7 months of age and kidding occurring between January and early February. During lactation, the diet was a standard forage-concentrate mix, with the major forage being alfalfa hay. Water was available ad libitum at all times. Milking was performed twice daily until October, followed by a 3-month dry period. At 4 months of age, all goats were vaccinated against Q fever (Coxevac, CEVA Santé Animale, France). At the time of the LPS challenge, all animals were primiparous in first lactation, approximately one year old and at 73.7 days in milk on average (DIM, sd = 9, min = 56, max = 96).

### Systemic inflammatory challenge and sampling protocol

The LPS challenge was performed on 83 goats from Facilities 1 and 2, in spring 2021 and spring 2023, respectively (Table 2). The LPS challenge followed the method previously described by [18]. One bolus dose of lipopolysaccharide (ultrapure **LPS** from *Escherichia coli*; InVivogen, Toulouse, France) of 0.5 µg/kg live weight was injected in the jugular vein by a veterinarian. The total amount of injected LPS was adjusted to the live weight based on the average of 2 weighings carried out in the previous week. Goats were briefly manually restrained in the common pen for blood collection, as they were habituated to handling. Before injection, the site of injection was disinfected with 70% alcohol. LPS was injected between 08:00 and 09:00 AM on the day of the challenge after morning milking.

**Table 2:** Distribution of the 83 goats within the 2 divergent lines selected on high longevity (High_LGV) or low longevity (Low_LGV) bred at two INRAE facilities that underwent in-vivo inflammatory challenge.

		Farm 1	Farm 2	*Total*
**Longevity line**	**Low_LGV**	18	23	41
	**High_LGV**	25	17	*42*
	***Total***	*43*	*40*	*83*

Rectal temperature was recorded at hours 4, 6, 8, 10, 12, 24, 48 and 72 in Facility 1, and at hours 0, 2, 4, 6, 8, 12, 24, 48 and 72 in Facility 2. Both schedules bracket the expected hyperthermia peak (~4–5 h post-injection [18,19]), so the maximum rectal temperature was captured in all goats. A digital thermometer was used and goats were briefly restrained manually. The occurrence of diarrhoea was also monitored as a binary observation (presence/absence of liquid faeces) at each sample time from 0 to 24 hours. The percentage of goats that experienced diarrhoea at least once was calculated (**%Dia**), the maximal rectal temperature (**maxTrec**), and the mean rectal temperature (**meanTrec**). The milk yield (**MY**) was measured at every morning milking on days −3, 0, 1, 2, 3, and 7 after the LPS injection, day 0 being the last morning milking before the LPS injection. The mean MY before challenge (**MY_bc**) based on MY at day −3 and 0 was calculated. **MY_min** corresponded to the minimum MY reached after LPS injection during days 1,2, and 3. The MY decrease after LPS injection was calculated as **MY_decrease** = 100*(MY_bc – MY_min)/MY_bc.

Blood was drawn by jugular venipuncture 12 times from each goat at -72h, 0h (injection time), 2h, 4h, 6h, 8h, 10h, 12h, 24h, 48h, 72h, and 144h. Hour 0 represents the blood sample taken just before the LPS injection. The blood samples were collected using Heparin-coated tubes and centrifuged at 2500 × g for 12 min at 4 °C within 15 minutes of collection so the plasma could be collected with a pipette, transferred to deep-well plates, and stored at −80 °C until further analysis.

Plasma samples were analysed with a commercially available bead-based multiplex immunoassay (MILLIPLEX® MAP Ovine Cytokine/Chemokine Panel 1, EMD Millipore Corp., Billerica, MA, USA) according to the manufacturer’s instructions. The concentrations of 14 cytokines were measured in: **IFN-γ** (Interferon-gamma), **IL-1α** (Interleukin-1 alpha), **IL-1β** (Interleukin-1 beta), **IL-4** (Interleukin-4), **IL-6** (Interleukin-6), **IL-10** (Interleukin-10), **CXCL8** (Interleukin-8), **IL-17A** (Interleukin-17 A), **MIP1α/CCL3** (Macrophage Inflammatory Protein-1 alpha), **IL-36RA** (Interleukin-36 Receptor Antagonist), **CXCL10** (Interferon-inducible Protein 10), **MIP1β/CCL4** (Macrophage Inflammatory Protein-1 beta), **TNFα** (Tumor Necrosis Factor alpha), **VEGFα** (Vascular Endothelial Growth Factor alpha). Concentration results were expressed as pg/mL. Caprine IL-6 was determined using a commercial ELISA following the manufacturer’s recommendations (KingFisher Biotech).

### Whole blood assay with lipopolysaccharide

The ex vivo whole blood assay (WBA) was performed on a larger cohort of 157 goats: at Facilities 1 and 2 at the same time as the in vivo challenge (spring 2021 and spring 2023, respectively), and at Facility 3 over two consecutive year-cohorts (spring 2021 and spring 2022. All challenges were conducted during the spring lactation period.

The whole blood assay involved direct blood sampling into syringes (S-Monovettes, Starstedt) previously filled with a 10X LPS solution giving a final LPS concentration of 3 µg/mL of whole blood (30 µg/mL, same source as in vivo experiment) that were placed on a dry bath (39°C, physiological core body temperature of the goat) for 24 hours as described by [20]. A parallel unstimulated control was collected without LPS to provide the baseline concentration. In facilities 1 and 2 where in vivo challenges were also conducted, the WBA was performed on a separate blood sample collected at the same time as the pre-challenge blood draw (hour 0).

Three variables were derived from the WBA: the baseline cytokine concentration without LPS stimulation (Conc_null), the cytokine concentration with LPS stimulation (Con_LPS) and the delta response (delta) that was calculated as the difference between stimulated and unstimulated samples, providing a measure of the reactivity and representing the net cytokine production specifically induced by the LPS challenge.

### Comparison of the response to the in vivo challenge between lines

All statistical analyses were performed in the R statistical environment (https://www.r-project.org/). Baseline cytokine concentrations were calculated for each animal as the mean concentration between hours −72 and 0 (pre-injection). The baseline concentrations between longevity lines for all 14 measured cytokines was compared using linear regression with the following model:


$$\begin{array}{c} \text{log}\left({\text{Baseline}\_\text{Concentration}}_{\text{jl}}\right)=\mu +\alpha_{\text{j}}+\gamma_{\text{l}}+\epsilon_{\text{jl}} \end{array}$$ (1)

where:

- Baseline_Concentration is the mean cytokine concentration at hours −72 and 0
- μ is the intercept
- α_j_ is the effect of the facility j
- γ_l_ is the effect of line l
- εjl ~ N(0, σ²ε) is the residual error

Statistical significance was assessed using ANOVA on the linear model, with p-values < 0.05 considered significant.

Among the 14 measured cytokines, only those showing a clear response to LPS injection were retained for the temporal comparison between longevity lines based on a non-overlap criterion: a cytokine was considered activated if the 95% confidence interval of the mean concentration at one or more post-injection time points did not overlap with the confidence interval at hour 0 and hour −72, and only when this occurred within a biologically coherent window (an increase during the expected post-injection period, rather than an isolated point or a faint, late drift).

Linear mixed regression was fitted to compare the cytokine time course between the two longevity lines using the following mixed model:


$$\begin{array}{c} \text{log}\left({\text{Concentration}}_{\text{ijkl}}\right)=\mu +\alpha_{\text{j}}+\tau_{\text{k}}+\gamma_{\text{l}(\text{k})}+{\text{u}}_{\text{i}}+\epsilon_{\text{ijkl}} \end{array}$$ (2)

where:

Concentration_ijkl_ is the cytokine concentration for animal i, Facility j, hour k and longevity line l

- μ is the intercept
- α_j_ is the effect of the facility j
- τ_k_ is the effect of time (hour k)
- γ_l(k)_ is the effect of line l nested within Hour k
- ui ~ N(0, σ²u) is the random effect of animal
- εijkl ~ N(0, σ²ε) is the residual error

The repeated measures structure was accounted for by the random animal intercept (u_i_), which captures the between-animal variability and induces a compound symmetry correlation structure among the repeated observations from the same individual. Hour was treated as a categorical fixed factor (12 levels), and the Line effect was nested within Hour, meaning that a separate Line contrast was estimated at each time point. The model was fitted using the lmer function from the lme4 package in R [21]. Post-hoc contrasts between lines at each time point were extracted using the emmeans package.

Clinical and production variables were compared between longevity lines using linear models (lm function in R), with Line as the fixed effect of interest and Facility included as a fixed effect to account for between-farm differences (two levels: facility 1 or facility 2). The variables tested were: the proportion of goats that showed diarrhoea at least once during the 24 hours following LPS injection (Dbinary outcome analysed as a proportion), the maximum rectal temperature reached during the challenge, the mean daily milk yield before LPS injection (averaged over days −3 and 0), the minimum daily milk yield observed during the two days following LPS injection, and the relative milk yield decrease after LPS injection (expressed as a percentage). The significance of the line effect was assessed using Type II ANOVA (car package), with p-values < 0.05 considered significant.

### Comparison of the response to the WBA between lines and correlation between in vivo and ex vivo challenge

The three ex vivo variables (Conc_null, Conc_LPS, and delta) were log-transformed to meet normality assumptions and compared between longevity lines among the 157 goats sampled across the four facility × year environments (Facility 1 in 2021, Facility 2 in 2023, Facility 3 in 2021, and Facility 3 in 2022). A linear model was fitted for each variable, with Line as the fixed effect of interest and the facility × year combination included as a fixed effect (4 levels) to account for environmental variation arising from differences in management, climate, and laboratory batch across facilities and years. The significance of the line effect was assessed using Type II ANOVA, with p-values < 0.05 considered statistically significant.


$$\begin{array}{c} \text{log}\left(\gamma_{\text{jl}}\right)=\mu +{\text{a}}_{\text{j}}+\gamma_{\text{l}}+\epsilon_{\text{jl}} \end{array}$$ (3)

where:

- Y_ij is the ex vivo variable (Conc_null, Conc_LPS, or delta) μ is the intercept
- μ is the intercept
- α_j_ is the effect of the environment j (4 levels: Facility 1 in 2021, Facility 2 in 2023, Facility 3 in 2021, Facility 3 in 2022)
- γ_l_ is the effect of line l
- εjl ~ N(0, σ²ε) is the residual error

To evaluate the predictive value of the ex vivo whole blood assay for in vivo responses, correlations were computed between the three ex vivo IL-6 variables (Conc_null, Conc_LPS, delta) and the in vivo IL-6 concentrations measured at each of the 12 time points (-72h to 144h) during the LPS challenge. This analysis was restricted to the 82 goats from Facilities 1 and 2 for which both in vivo and ex vivo measurements were available. Pearson correlation coefficients were calculated, and their statistical significance was assessed with the cor.test function in R. This approach allowed us to identify the time points at which ex vivo reactivity most strongly predicted the in vivo inflammatory response.

## Results

### Description of the response to LPS injection

No animals reached humane endpoint criteria, and none were euthanized after LPS injection. One goat died during the experiment; post-mortem examination revealed extensive fibrinous pleuropneumonia with no other lesions, indicating a pre-existing condition whose progression may have been precipitated by the LPS challenge. This goat was removed from the analysis and is not counted in Table 1 and Table 2.

**Fig 1:**
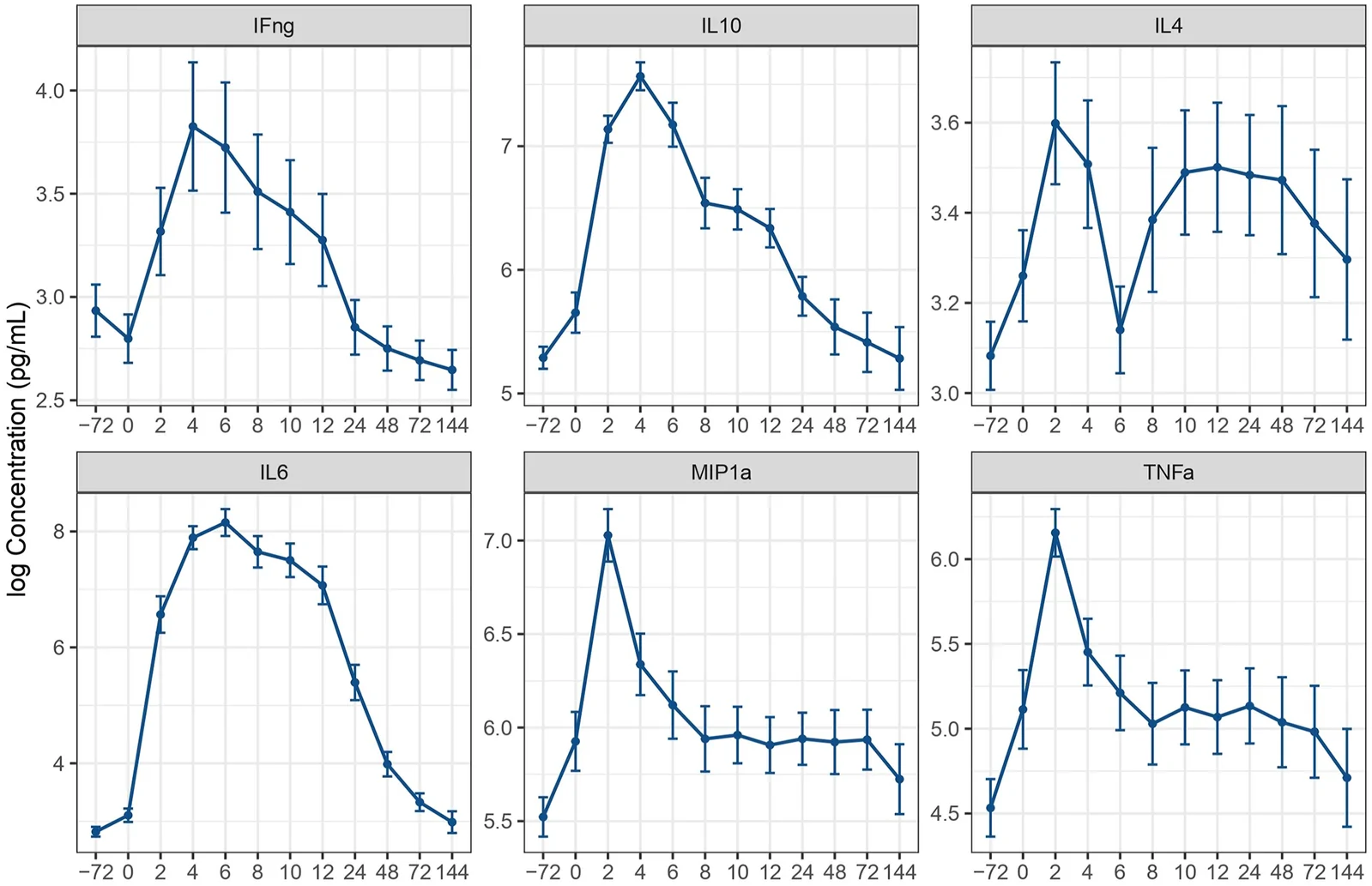
Clinical and production responses to LPS injection in 83 Alpine dairy goats. (A) Proportion of goats showing diarrhoea at each monitoring time point from hour 0 to hour 24; error bars represent 95% Wald confidence intervals for binomial proportions. (B) Mean rectal temperature (°C) measured from hour 0 to hour 72; error bars indicate 95% confidence intervals (mean ± 1.96 × SD/√n). (C) Mean daily morning milk yield (kg) from day 0 to day 7 relative to LPS injection; error bars as in panel B. Hour 0 and day 0 correspond to the last measurements taken before LPS injection. Detailed values are available in supplementary data.

The clinical and production responses to LPS injection are shown in Fig 1. Rectal temperature rose sharply from a mean of 38.6°C (95% CI: 38.4–38.7) at hour 0 to a peak of 40.6°C (95% CI: 40.5–40.8) at hour 4, representing a mean increase of 2.1°C (S1 Table). Temperature then gradually declined, reaching 39.1°C (95% CI: 39.0–39.2) at hour 24 and returning close to baseline by hour 72 (38.8°C, 95% CI: 38.7–38.9). The proportion of goats showing diarrhoea increased from 0% at hour 0 to a maximum of 24.1% (95% CI: 14.9–33.3%) at hour 8, before declining to 11.0% (95% CI: 4.2–17.7%) at hour 24 (S2 Table). Over the first 24 hours post-injection, 34% of goats experienced diarrhoea at least once (Table 3). The mean morning milk yield at day −3 was 1.9 kg. Milk yield dropped to a minimum of 1.6 kg (95% CI: 1.5–1.7) on day 1 after injection, corresponding to a 16.4% decrease relative to day −3, before progressively recovering to 1.8 kg (95% CI: 1.7–1.9) by day 3 and 1.9 kg (95% CI: 1.7–2.0) by day 15 (S3 Table).

**Table 3:** Comparison of clinical and production variables between the two longevity lines (High_LGV and Low_LGV) among the 83 goats that underwent the in vivo challenge. P-values correspond to the line effect in the linear model correctied for facility.

	Mean (n = 83)	Longevity line		
		High_LGV (n = 42)	Low_LGV (n = 41)	p
Dia (%)	34.0	38.0	29.0	0.641
maxTrec (°c)	40.7	40.7	40.8	0.520
MY_bc (kg)	1.9	1.8	2.0	0.084
MY_min (kg)	1.3	1.3	1.4	0.453
MY_decrease (%)	30.9	30.5	31.2	0.929

(***) p < 0.001, (**) 0.001 < p < 0.01, (*) 0.01 < p < 0.05, (.) 0.05 < p < 0.1

Dia: the percentage of goats who had diarrhoea at least once during the day after LPS injection; maxTrec: maximum body temperature; MY_bc: milk yield before LPS injection; MY_min: minimum milk yield reached during the two days following LPS injection; MY_decrease: percentage of milk yield decrease after LPS injection.

**Fig 2:**
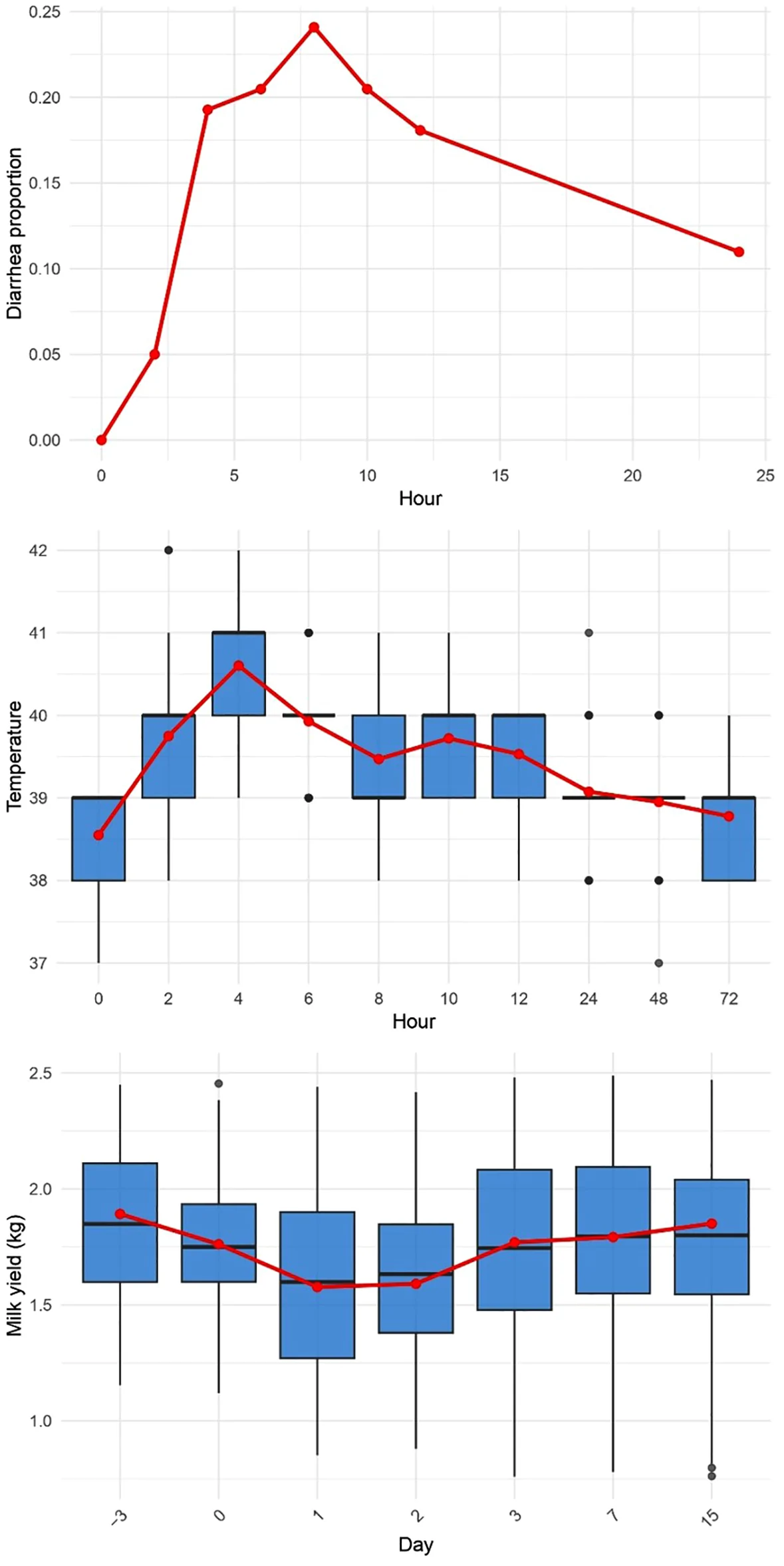
The mean curves of the blood concentration (log transformed, in pg/ml) of 6 cytokines from 83 alpine dairy goats. Hour 0 corresponds to the intravenous injection of LPS. The vertical bars correspond to the 95% confidence interval ($mean\pm \frac{\text{1}.\text{9}\text{6}*SD}{\sqrt{n})}$). The 14 cytokines are **IFN-γ** (Interferon-gamma), **IL-4** (Interleukin-4), **IL-6** (Interleukin-6), **IL-10** (Interleukin-10), **MIP1α** (Macrophage Inflammatory Protein-1 alpha) and **TNFα** (Tumor Necrosis Factor alpha).

Among the 14 measured cytokines, six were identified as responsive to LPS injection: IL-6, IL-10, IFN-γ, TNFα, MIP1α, and IL-4. Among them, IL-6 showed the strongest and most sustained response, rising from a baseline of 16.8 pg/mL at hour −72 to a peak of 3463.8 pg/mL at hour 6, corresponding to an approximate 200-fold increase in concentration, before gradually returning toward baseline by hour 144 (19.9 pg/mL). IL-10 displayed a rapid increase from 198.4 pg/mL at hour −72 to a peak of 1939.1 pg/mL at hour 4, followed by a progressive decline. IFN-γ exhibited a more moderate response, peaking at 46.1 pg/mL at hour 4 and returning close to baseline by hour 24. TNFα and MIP1α both showed sharp but transient peaks at hour 2 (473.4 and 1130.0 pg/mL, respectively), with concentrations returning close to pre-challenge levels by hour 4–6. IL-4 showed the most modest response, with a slight increase from 21.8 pg/mL at hour −72 to 36.6 pg/mL at hour 2, followed by relatively stable concentrations. For all six cytokines, the variability among individuals was substantial, as reflected by the wide confidence intervals during the peak response period (Fig 2 and S4 Table). The concentrations at each time point of the eight cytokines that did not respond to LPS injection are presented in S5 Table.

### Comparison of the immune reaction to LPS injection between the two longevity lines

Table 4 shows the basal blood concentrations of the 14 measured cytokines, calculated as the mean of the concentrations measured at hours −72 and 0 before the LPS challenge. The baseline cytokine concentrations were compared between longevity lines for all 14 measured cytokines (Table 4). After correcting for the facility effect, no significant differences were observed between High_LGV and Low_LGV goats for any cytokine. However, near-significant trends were observed for MIP1α (p = 0.06) and CXCL10 (p = 0.08) for which the High_LGV goats had greater blood basal concentrations.

**Table 4:** Baseline cytokine concentrations (mean of hours −72 and 0) in the two longevity lines of goats (High_LGV and Low_LGV) among the 83 goats that underwent the in vivo challenge. Concentrations are expressed in pg/mL. The p-value corresponds to the significance of the line effect in the linear regression model, correcting for facility effect.

Cytokine	Mean basal concentration in pg/mL (sd)		p_value
	High_LGV	Low_LGV	
CXCL10	4565.8 (1875.28)	4409.4 (1678.9)	0.085
CXCL8	10878.6 (5876.74)	10870.4 (4702.96)	0.109
IFng	19.5 (10.35)	24.8 (29.4)	0.431
IL10	335.4 (208.64)	273.1 (213.5)	0.255
IL17a	18.4 (12.37)	16.4 (9.18)	0.408
IL1a	309.7 (246.46)	245.1 (285.35)	0.255
IL1b	14.6 (9.67)	13.9 (8.74)	0.794
IL36RA	1247.6 (695.83)	1025.4 (739.66)	0.180
IL4	27.9 (12.59)	24.7 (9.34)	0.464
IL6	22.6 (12.22)	22.0 (9.27)	0.237
MIP1a	436.1 (240.37)	333.8 (233.44)	0.067
MIP1b	29.9 (15.29)	28.2 (17.08)	0.417
TNFa	238.3 (199.3)	184.5 (220.03)	0.196
VEGFa	890.1 (441.9)	754.3 (569.4)	0.237

**Fig 3:**
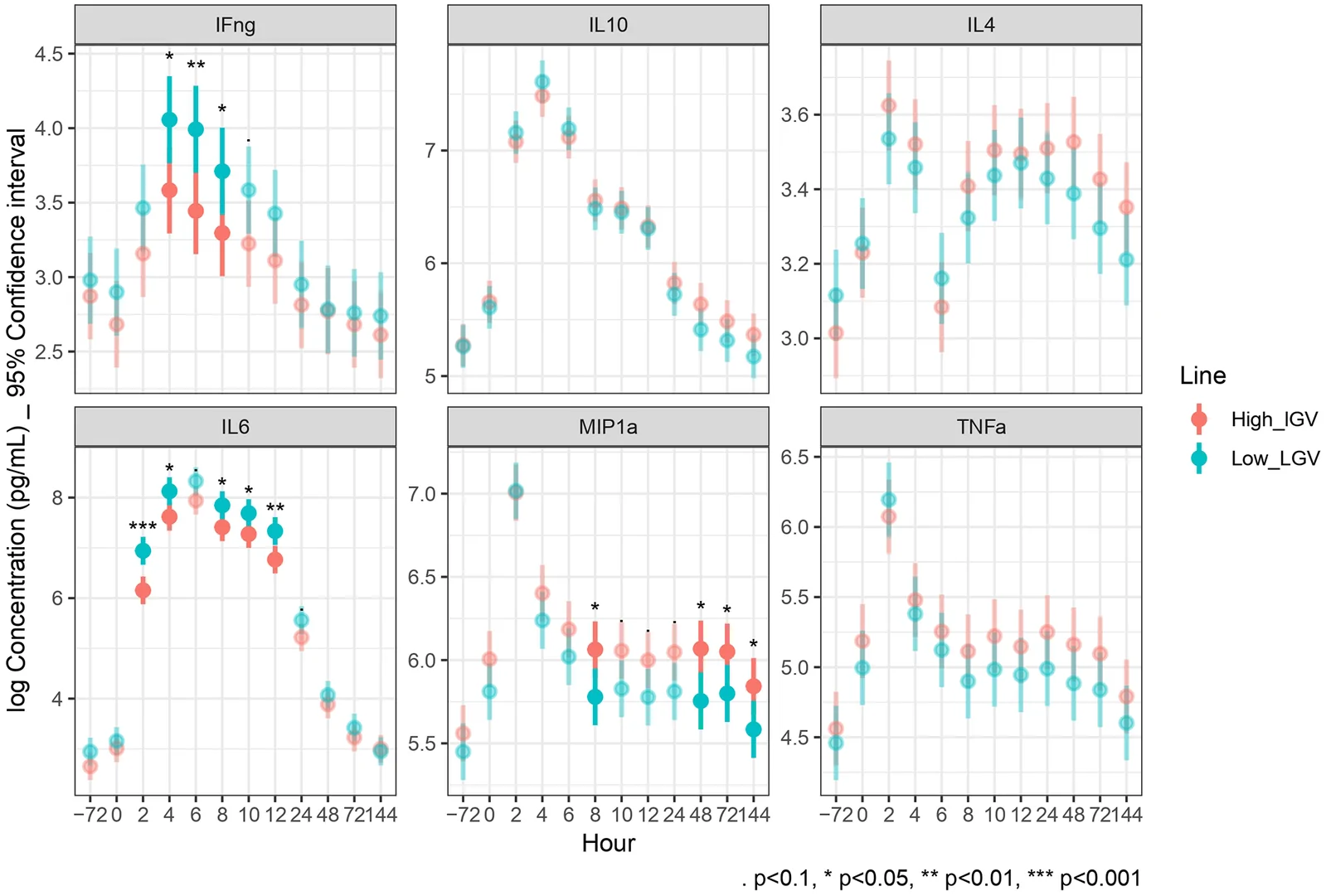
Comparison of the log(concentration) of 6 cytokines after injection of LPS (h = 0) between the two longevity lines (High_LGV, and Low_LGV). A mixed model was fitted, including an animal random effect, a line effect nested in the hour and a fixed facility effect. The log(Concentration) of the cytokines was compared. The figure shows the average least squares means estimates and confidence interval for each line at each time point. The transparent color corresponds to time points when the difference is not significant between lines (p > 0.05).

Among the 14 measured cytokines, six showed a clear response to LPS injection, and were retained for temporal comparison between longevity lines: IFN-γ, IL-4, IL-6, IL-10, MIP1α/CCL3, and TNFα. The outcomes of the mixed models used to compare the cytokine concentrations between lines at each time point are shown in Fig 3 (95% confidence interval of estimated mean per line and p-value) and S6 Table. Three of the six LPS-responsive cytokines showed significant differences between longevity lines. The Low_LGV goats demonstrated a greater acute inflammatory response, with significantly greater concentrations of IFN-γ and IL-6 during the early hours post-LPS injection (hours 4–8 and 2–12, respectively). These differences disappeared at later time points as concentrations returned to baseline levels. In contrast, the High_LGV line displayed significantly greater MIP1α concentration after 8h post-injection. IL-10, IL-4, and TNFα concentrations showed no significant differences between lines across the measured time points despite their clear response to LPS stimulation.

### Comparison of WBA results between longevity lines

ANOVA analysis revealed no significant differences between the two longevity lines (after accounting for facility x year effects) for any of the three ex vivo IL-6 variables examined (Table 5). Baseline IL-6 cytokine concentrations without LPS stimulation (Conc_null) showed mean values of 4.5 ng/mL for High_LGV and 4.4 ng/mL for Low_LGV goats (p = 0.79). Similarly, LPS-stimulated concentrations (Conc_LPS) were comparable between lines, as were the delta responses representing net LPS-induced IL-6 production.

**Table 5:** Results of the IL-6 concentration (in ng/mL) in the ex vivo challenge among the 157 goats from the two longevity lines (High_LGV and Low_LGV). The p-value corresponds to the significance of the line effect in the ANOVA.

	Mean (CI) (n = 157)	Longevity line		
		High_LGV (n = 84)	Low_LGV (n = 74)	p
Conc_null	4.5 (1.46)	4.5 (2.09)	4.4 (2.07)	0.794
Conc_LPS	7.8 (1.89)	8.3 (2.22)	7.2 (2.86)	0.721
Delta	3.4 (0.83)	3.9 (0.54)	2.8 (1.50)	0.836

### Correlation between ex vivo and in vivo immune responses

**Fig 4:**
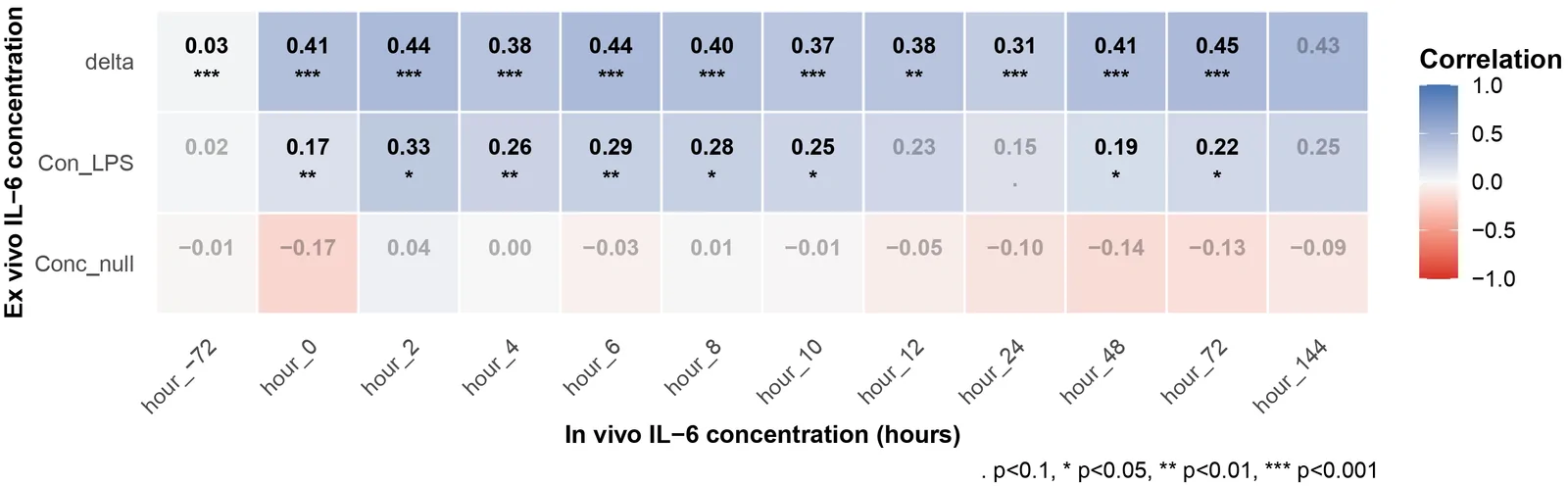
Correlation matrices between ex vivo and in vivo IL-6 responses in facilities 1 and 2 (83 goats). Pearson correlation coefficients between ex vivo immune variables (Conc_null: baseline unstimulated concentration; Conc_LPS: LPS-stimulated concentration; delta: stimulated minus unstimulated difference) and in vivo cytokine concentrations measured at multiple time points (-72h to 144h) during the LPS challenge. Color intensity represents correlation strength (purple: positive; orange: negative). Only statistically significant correlations (p < 0.05) are bolded.

Correlations between WBA variables and in vivo IL-6 concentrations measured at multiple time points throughout the LPS challenge were analysed in facilities 1 and 2 (Fig 4). The ex vivo concentration showed moderate to strong correlations with the in vivo response. IL-6 displayed positive correlations between ex vivo measurements (particularly Conc_LPS and delta) and in vivo concentrations during the early to mid-challenge period (Fig 4), with the strongest associations observed between 0h and 72h post-challenge (r ranging from 0.30 to 0.51 for this time period).

## Discussion

Our study aimed to explore the underlying mechanisms of resilience in dairy goats, using functional longevity as a key selection criterion. Functional longevity reflects an animal’s capacity to remain productive over its lifespan, which inherently depends on its ability to overcome and recover from the various environmental perturbations encountered during its productive life. Among these perturbations, infectious diseases represent a major challenge. Indeed, Sasaki and Rostellato et al. [7,22] demonstrated that health events, particularly mastitis, are among the most explanatory factors of functional longevity in dairy ruminants. Hence, the hypothesis was made that genetic selection for divergent functional longevity would result in different immune response profiles between the High_LGV and Low_LGV lines when confronted with an inflammatory challenge. Our results partially support this hypothesis. Furthermore, in high-yielding animals, digestive tract disorders, impacting and impacted by systemic inflammation, can significantly impair liver function and overall health, thereby affecting functional longevity [23]. Notably, using the same longevity lines submitted to a 2-day underfeeding challenge, Ithurbide et al. [24] showed that goats with the strongest metabolic disturbance during feed restriction had poorer survival. That study also revealed a link between nutritional stress and inflammation, as the underfeeding challenge triggered an increase in milk LDH — an indicator of mammary inflammation — that was more prolonged in the least resilient animals. Together with the present findings on innate immune reactivity, these results suggest that both metabolic and inflammatory dimensions contribute to the heritable mechanisms underlying functional longevity in dairy goats.

The blood concentrations of 6 of the 14 measured cytokines changed over time after the LPS injection. Indeed, IFN-γ, IL-4, IL-6, IL-10, TNFα, and MIP-1α showed clear increased concentration in the hours following LPS injection. Pelayo et al. [12] also showed that IL-6 had the highest increase amongst the measured cytokines after intra-mammary LPS injection in ewes at 6 hours post-challenge. The early increase of the anti-inflammatory cytokines IL-10 and IL-4 that reached a maximum at hour 4 after challenge in our study is also consistent with the literature data [12,19]. However, Pelayo et al. [12] showed no increase of IFN-γ, MIP1α and TNFα. As in the present study, Pelayo et al. [12] showed no increase of IL-1α, IL-1β, and IL-17A after LPS injection. The consistency of these dynamics with ovine data also supports applying the multiplex assay to goats. Although not validated by the manufacturer for caprine plasma, this panel was developed with Merck as a cross-ruminant platform, initially validated in bovine samples [20], with reagents selected for their cross-reactivity and the high sequence conservation of the targeted cytokines across ruminants; to our knowledge, this is the first application to caprine samples. IL-6 was independently confirmed with a caprine-specific ELISA (KingFisher Biotech), with consistent results. As a formal analytical validation (parallelism, spike-and-recovery, dilution linearity) was not feasible given the scarcity of recombinant caprine cytokines, the values should be read as relative rather than absolute concentrations, suitable for comparing cytokine profiles across experimental groups and time points.

While no differences in baseline cytokine concentrations were observed between lines, three of the six cytokines that responded to LPS injection showed significantly different temporal profiles between longevity lines. Low_LGV goats responded more intensely to the challenge with greater IFN-γ and IL-6 production during the early hours post-injection, whereas High_LGV goats exhibited greater concentrations of MIP1α during the later phase of the inflammatory response.

IL-6 is a pro-inflammatory cytokine that drives stereotypical responses to infection, such as fever [19]. Masaoki et al. [25] proposed that IL-6-mediated increased synthesis of angiotensinogen in the liver after induction of inflammation. IL-6 was identified as the primary inducer of hepatic synthesis of acute-phase proteins [26]. Moreover, the inflammatory induction of positive acute-phase proteins by IL-6 and other pro-inflammatory cytokines is accompanied by a concurrent reduction in negative acute-phase proteins such as lipoproteins, and paraoxonase, reflecting impaired liver functionality that may contribute to reduced functional longevity [23]. Kaplanski et al. [27] proposed that IL-6 regulates the transition to leukocyte recruitment through a shift of chemokine production, and thus is a key regulator of acute inflammation. Again, IL-6 is recognized as a reliable indicator of subclinical mastitis in dairy cows [26,28]. It was also described as an early indicator of subclinical endometritis in cattle [29,30]. IFN-γ is a critical pro-inflammatory cytokine primarily produced by natural killer cells and T lymphocytes, playing a central role in both innate and adaptive immune responses. It activates macrophages, enhances antigen presentation, promotes the differentiation of Type 1 helper cells, and stimulates the production of other pro-inflammatory mediators. The elevated IFN-γ response in Low_LGV goats suggests a more pronounced activation of cell-mediated immunity and heightened inflammatory reactivity, which aligns with the overall pattern of stronger acute-phase responses observed in this line. MIP1α (also known as CCL3) is a chemokine involved in leukocyte recruitment and activation, playing important roles in orchestrating the resolution phase of inflammation by regulating immune cell trafficking and promoting tissue repair.

The observation that baseline cytokine concentrations did not differ between longevity lines, while the LPS challenge revealed significant differences in response dynamics, resonates with recent conceptual frameworks for phenotyping resilience in livestock. Friggens et al. and Ithurbide et al. [31,32] emphasised the necessity of a challenge or perturbation to reveal the underlying resilience mechanisms of an animal. In the present study, this principle is clearly illustrated: the differences between longevity lines only became apparent when animals were subjected to the LPS challenge, remaining undetectable under baseline conditions. Furthermore, Friggens et al. [31] highlighted the dynamic nature of resilience and the need for longitudinal data to reveal the elastic response of the organism to short-term environmental perturbations. Here again, the repeated measurements across twelve time points allowed us to capture the distinct temporal dynamics between lines and cytokines. Notably, the differences between lines where High_LGV goats showed greater MIP1α concentration occurred during the resolution phase of the inflammatory process, whereas Low_LGV goats exhibited stronger IFN-γ and IL-6 responses during the peak of acute inflammation. This temporal pattern suggests that High_LGV goats may be characterised not by a blunted initial response but rather by enhanced regulatory mechanisms and more efficient resolution processes, which could contribute to faster recovery and reduced tissue damage following inflammatory challenges.

The acute-phase reaction to systemic disturbances leads to a rapid increase in the production of proteins, recognised as markers of inflammation [33,34]. In the present study, the intravenous bolus of LPS induced a systemic response as evidenced by measurement of the rectal temperature, diarrhoea, and blood concentrations of a large cytokine/chemokine panel. Specifically, the LPS injection induced hyperthermia in the hours following administration, reaching a maximum at 4 hours post-injection. This aligns with results reported in female lambs subjected to an intravenous LPS challenge [18,19]. Acute phase inflammation can cause diarrhoea because of increased intestinal permeability and motility stimulated by inflammatory mediators, and altered fluid balance [18–20]. In the present study, 34% of goats had diarrhoea in the hours following LPS injection. Despite significant differences in cytokine profiles between longevity lines, no significant differences was observed in clinical manifestations, including hyperthermia, diarrhoea, or milk yield drop (Table 3). This likely reflects that production traits are influenced by multiple physiological systems beyond immunity, potentially masking immune-specific contributions in this single-challenge experimental context. Indeed, while statistically significant differences in cytokine profiles were detected between lines, the immune responses were not sharply contrasted between the two groups, with considerable overlap in individual trajectories. This outcome is somewhat expected given the nature of the divergent selection criterion used to create the two lines. Functional longevity is inherently a “black box” trait, resulting from the integration of numerous underlying mechanisms that can contribute to extended or shortened productive lifespan. Beyond immune competence, factors such as resistance to lameness, milking temperament and behaviour, energetic metabolism [24], and reproductive performance all contribute to an animal’s ability to remain in the herd. Consequently, it is not surprising that two sharply distinct immune trajectories were not observed between longevity lines. Nevertheless, the fact that, on average, certain cytokine profiles differed significantly between High_LGV and Low_LGV goats supports the hypothesis that immune reactivity is among the mechanisms that have been indirectly mobilized through selection on functional longevity. This finding reinforces the notion that immunity constitutes one component—albeit not the sole determinant—of the complex biological architecture underlying animal resilience and longevity.

An important aspect of our experimental design was the inclusion of multiple environments across the four facilities x year, which allowed us to assess the longevity line differences under varying conditions. This multi-environment approach substantially strengthened the biological relevance of our findings by testing whether genetic effects persisted across diverse environmental contexts. Genotype-by-environment (G × E) interactions are commonly reported in animal genetics, meaning that the relative advantage of one genotype over another may differ across environments. Environmental factors are known to significantly impact immune responses through multiple mechanisms, including differences in pathogen exposure, sanitary conditions [26], ventilation quality [35–37], and management practices such as feeding systems [12]. These environmental influences can alter both baseline inflammatory status and reactivity to immune challenges. To account for this environmental variation and identify genetic differences that were consistent across contexts, the facility x year as a fixed effect was included in our ANOVA models. The facility effect captures an aggregate of multiple environmental differences — including feeding systems, stocking density, and local pathogen pressure — and to attribute this effect to any single factor is not possible with the present design. Nevertheless, this multi-environment approach is more informative than a single-facility study, as it favours the detection of genetic effects that are likely to hold across a range of commercial farming conditions.

The WBA was conducted to evaluate whether a simplified test could predict individual immune responses observed during the in vivo challenge, which would offer a less invasive and more practical approach for assessing immune reactivity in breeding programs. Our results revealed no significant differences for the measured IL-6 cytokine of the ex vivo response between the two longevity lines, in contrast with the differences that were observed during the in vivo challenge. This discrepancy suggests that the complex physiological and systemic regulatory mechanisms involved at the organism scale may not be fully captured in the blood assay, where isolated blood cells respond to LPS stimulation outside their natural tissue context. In particular, the role and secretion of endothelial cells are not present, and the cytokine-cytokine circuitry among different tissues and organs is absent. The correlation analyses between ex vivo and in vivo responses showed a moderate predictive value. Correlations between ex vivo reactivity (delta) and in vivo concentrations of IL-6 were significantly different from hour 0 to hour 72 and ranged from 0.31 to 0.45, indicating that ex vivo testing may capture aspects of early responsiveness for these specific mediators. These findings highlight both the potential and limitations of an ex vivo challenge as a predictive tool. While the ex vivo approach offers practical advantages, including reduced animal stress, lower costs, and the ability to screen larger populations, it does not appear to capture the full complexity of in vivo immune responses.

## Conclusion

This study provides evidence that divergent selection for functional longevity in dairy goats is associated with differences in innate immune response profiles following a lipopolysaccharide challenge. While baseline cytokine concentrations were similar between lines, three of the six LPS-responsive cytokines showed distinct temporal patterns. Low longevity goats tended to exhibit a stronger acute inflammatory response, with greater IL-6 and IFN-γ concentrations during the early hours post-challenge, whereas high longevity goats showed elevated MIP1α concentration during the resolution phase. These observations suggest that innate immune reactivity may contribute, at least in part, to the heritable mechanisms underlying functional longevity in dairy goats, although other physiological systems are also likely involved.

## Supporting information

S1 Table Mean rectal temperature (°C) at each time point from hour 0 to hour 72 following intravenous LPS injection in 83 Alpine dairy goats. Values are presented as mean, standard deviation (SD), and 95% confidence interval of the mean (CI).
(DOCX)

S2 Table Proportion of goats showing diarrhoea at each monitoring time point from hour 0 to hour 24 following intravenous LPS injection in 83 Alpine dairy goats. Values are presented as proportion, with lower and upper bounds of the 95% Wald confidence interval.
(DOCX)

S3 Table Mean daily morning milk yield (kg) from day 0 to day 7 relative to LPS injection in 83 Alpine dairy goats. Values are presented as mean, standard deviation (SD), and 95% confidence interval of the mean (CI).
(DOCX)

S4 Table Mean log-transformed concentrations (ln pg/mL) of the six LPS-responsive cytokines (IFN-γ, IL-4, IL-6, IL-10, MIP1α, TNFα) at each time point from hour −72 to hour 144 relative to LPS injection in 83 Alpine dairy goats. Values are presented as mean, standard deviation (SD), and 95% confidence interval of the mean (CI = 1.96 × SD/√n).
(DOCX)

S5 Table Mean log-transformed concentrations (ln pg/mL) of the eight cytokines not responsive to LPS injection (CXCL10, CXCL8, IL-17a, IL-1α, IL-1β, IL-36RA, MIP1β, VEGFα) at each time point from hour −72 to hour 144 in 83 Alpine dairy goats. Values are presented as mean, standard deviation (SD), and 95% confidence interval of the mean (CI = 1.96 × SD/√n).
(DOCX)

S6 Table Comparison of log-transformed cytokine concentrations (ln pg/mL) between the two longevity lines (High_LGV and Low_LGV) at each time point from hour −72 to hour 144 following LPS injection in 83 Alpine dairy goats. Values are least squares means and 95% confidence intervals estimated from the mixed model [2], correcting for facility effect and including a random animal intercept. P-values correspond to the line contrast at each time point, extracted using the emmeans package. Significance levels: (***) p < 0.001, (**) p < 0.01, (*) p < 0.05, (.) 0.05 < p < 0.10.
(DOCX)
