# Supplementary material for: Effect of longevity genotype on acute phase response to a lipopolysaccharide challenge in dairy goats
Source: PLoS One. 2026 Aug 28;21(8):e0356779. doi: 10.1371/journal.pone.0356779 (PMC13524232; doi:10.1371/journal.pone.0356779)
Supplement: Supplementary file 1 [file pone.0356779.s001.docx]

**Supp table 1.** Mean rectal temperature (°C) at each time point from hour 0 to hour 72 following intravenous LPS injection in 83 Alpine dairy goats. Values are presented as mean, standard deviation (SD), and 95% confidence interval of the mean (CI).

| **Hour** | **N** | **Mean** | **SD** | **95% CI** |
| --- | --- | --- | --- | --- |
| 0 | 40 | 38.6 | 0.55 | 0.17 |
| 2 | 40 | 39.8 | 0.81 | 0.25 |
| 4 | 83 | 40.6 | 0.71 | 0.15 |
| 6 | 83 | 39.9 | 0.62 | 0.13 |
| 8 | 83 | 39.5 | 0.55 | 0.12 |
| 10 | 43 | 39.7 | 0.5 | 0.15 |
| 12 | 83 | 39.5 | 0.53 | 0.11 |
| 24 | 81 | 39.1 | 0.49 | 0.11 |
| 48 | 81 | 40.0 | 0.42 | 0.09 |
| 72 | 81 | 38.8 | 0.5 | 0.11 |
