# Supplementary material for: Effect of longevity genotype on acute phase response to a lipopolysaccharide challenge in dairy goats
Source: PLoS One. 2026 Aug 28;21(8):e0356779. doi: 10.1371/journal.pone.0356779 (PMC13524232; doi:10.1371/journal.pone.0356779)
Supplement: Supplementary file 2 [file pone.0356779.s002.docx]

**Supp table 2.** Proportion of goats showing diarrhoea at each monitoring time point from hour 0 to hour 24 following intravenous LPS injection in 83 Alpine dairy goats. Values are presented as proportion, with lower and upper bounds of the 95% Wald confidence interval.

| **Hour** | **N** | **Proportion** | **CI lower** | **CI upper** |
| --- | --- | --- | --- | --- |
| 0 | 40 | 0.00 | 0.00 | 0.00 |
| 2 | 40 | 0.05 | -0.02 | 0.12 |
| 4 | 83 | 0.19 | 0.11 | 0.28 |
| 6 | 83 | 0.21 | 0.12 | 0.29 |
| 8 | 83 | 0.24 | 0.15 | 0.33 |
| 10 | 83 | 0.21 | 0.12 | 0.29 |
| 12 | 83 | 0.18 | 0.10 | 0.26 |
| 24 | 82 | 0.11 | 0.04 | 0.18 |
