# Supplementary material for: Effect of longevity genotype on acute phase response to a lipopolysaccharide challenge in dairy goats
Source: PLoS One. 2026 Aug 28;21(8):e0356779. doi: 10.1371/journal.pone.0356779 (PMC13524232; doi:10.1371/journal.pone.0356779)
Supplement: Supplementary file 3 [file pone.0356779.s003.docx]

**Supp table 3.** Mean daily morning milk yield (kg) from day 0 to day 7 relative to LPS injection in 83 Alpine dairy goats. Values are presented as mean, standard deviation (SD), and 95% confidence interval of the mean (CI).

| **Day** | **N** | **Mean** | **SD** | **95% CI** |
| --- | --- | --- | --- | --- |
| -3 | 86 | 1.89 | 0.35 | 0.07 |
| 0 | 86 | 1.76 | 0.33 | 0.07 |
| 1 | 86 | 1.58 | 0.51 | 0.11 |
| 2 | 86 | 1.59 | 0.42 | 0.09 |
| 3 | 86 | 1.77 | 0.52 | 0.11 |
| 7 | 86 | 1.79 | 0.47 | 0.1 |
| 15 | 86 | 1.85 | 0.54 | 0.11 |
