# Supplementary material for: Effect of longevity genotype on acute phase response to a lipopolysaccharide challenge in dairy goats
Source: PLoS One. 2026 Aug 28;21(8):e0356779. doi: 10.1371/journal.pone.0356779 (PMC13524232; doi:10.1371/journal.pone.0356779)
Supplement: Supplementary file 4 [file pone.0356779.s004.docx]

**Supp table 4.** Mean log-transformed concentrations (ln pg/mL) of the six LPS-responsive cytokines (IFN-γ, IL-4, IL-6, IL-10, MIP1α, TNFα) at each time point from hour -72 to hour 144 relative to LPS injection in 83 Alpine dairy goats. Values are presented as mean, standard deviation (SD), and 95% confidence interval of the mean (CI = 1.96 × SD/√n).

| **Cytokine** | **Hour** | **Mean** | **SD** | **95% CI** |
| --- | --- | --- | --- | --- |
| IFng | -72 | 2.9 | 0.59 | 0.13 |
| IFng | 0 | 2.8 | 0.54 | 0.12 |
| IFng | 2 | 3.3 | 0.98 | 0.21 |
| IFng | 4 | 3.8 | 1.44 | 0.31 |
| IFng | 6 | 3.7 | 1.47 | 0.32 |
| IFng | 8 | 3.5 | 1.29 | 0.28 |
| IFng | 10 | 3.4 | 1.17 | 0.25 |
| IFng | 12 | 3.3 | 1.04 | 0.22 |
| IFng | 24 | 2.9 | 0.62 | 0.13 |
| IFng | 48 | 2.8 | 0.50 | 0.11 |
| IFng | 72 | 2.7 | 0.44 | 0.10 |
| IFng | 144 | 2.6 | 0.45 | 0.10 |
| IL10 | -72 | 5.3 | 0.41 | 0.09 |
| IL10 | 0 | 5.7 | 0.76 | 0.16 |
| IL10 | 2 | 7.1 | 0.51 | 0.11 |
| IL10 | 4 | 7.6 | 0.52 | 0.11 |
| IL10 | 6 | 7.2 | 0.83 | 0.18 |
| IL10 | 8 | 6.5 | 0.95 | 0.20 |
| IL10 | 10 | 6.5 | 0.76 | 0.16 |
| IL10 | 12 | 6.3 | 0.72 | 0.16 |
| IL10 | 24 | 5.8 | 0.73 | 0.16 |
| IL10 | 48 | 5.5 | 1.03 | 0.22 |
| IL10 | 72 | 5.4 | 1.12 | 0.24 |
| IL10 | 144 | 5.3 | 1.18 | 0.25 |
| IL4 | -72 | 3.1 | 0.35 | 0.08 |
| IL4 | 0 | 3.3 | 0.47 | 0.10 |
| IL4 | 2 | 3.6 | 0.63 | 0.14 |
| IL4 | 4 | 3.5 | 0.66 | 0.14 |
| IL4 | 6 | 3.1 | 0.45 | 0.10 |
| IL4 | 8 | 3.4 | 0.74 | 0.16 |
| IL4 | 10 | 3.5 | 0.64 | 0.14 |
| IL4 | 12 | 3.5 | 0.67 | 0.14 |
| IL4 | 24 | 3.5 | 0.62 | 0.13 |
| IL4 | 48 | 3.5 | 0.76 | 0.16 |
| IL4 | 72 | 3.4 | 0.76 | 0.16 |
| IL4 | 144 | 3.3 | 0.83 | 0.18 |
| IL6 | -72 | 2.8 | 0.39 | 0.08 |
| IL6 | 0 | 3.1 | 0.54 | 0.12 |
| IL6 | 2 | 6.6 | 1.46 | 0.32 |
| IL6 | 4 | 7.9 | 0.92 | 0.20 |
| IL6 | 6 | 8.2 | 1.08 | 0.23 |
| IL6 | 8 | 7.6 | 1.26 | 0.27 |
| IL6 | 10 | 7.5 | 1.35 | 0.29 |
| IL6 | 12 | 7.1 | 1.51 | 0.33 |
| IL6 | 24 | 5.4 | 1.41 | 0.30 |
| IL6 | 48 | 4.0 | 0.98 | 0.21 |
| IL6 | 72 | 3.3 | 0.71 | 0.15 |
| IL6 | 144 | 3.0 | 0.87 | 0.19 |
| MIP1a | -72 | 5.5 | 0.49 | 0.11 |
| MIP1a | 0 | 5.9 | 0.73 | 0.16 |
| MIP1a | 2 | 7.0 | 0.65 | 0.14 |
| MIP1a | 4 | 6.3 | 0.76 | 0.16 |
| MIP1a | 6 | 6.1 | 0.84 | 0.18 |
| MIP1a | 8 | 5.9 | 0.81 | 0.18 |
| MIP1a | 10 | 6.0 | 0.70 | 0.15 |
| MIP1a | 12 | 5.9 | 0.69 | 0.15 |
| MIP1a | 24 | 5.9 | 0.65 | 0.14 |
| MIP1a | 48 | 5.9 | 0.80 | 0.17 |
| MIP1a | 72 | 5.9 | 0.74 | 0.16 |
| MIP1a | 144 | 5.7 | 0.87 | 0.19 |
| TNFa | -72 | 4.5 | 0.79 | 0.17 |
| TNFa | 0 | 5.1 | 1.08 | 0.23 |
| TNFa | 2 | 6.2 | 0.65 | 0.14 |
| TNFa | 4 | 5.5 | 0.91 | 0.20 |
| TNFa | 6 | 5.2 | 1.02 | 0.22 |
| TNFa | 8 | 5.0 | 1.12 | 0.24 |
| TNFa | 10 | 5.1 | 1.01 | 0.22 |
| TNFa | 12 | 5.1 | 1.01 | 0.22 |
| TNFa | 24 | 5.1 | 1.03 | 0.22 |
| TNFa | 48 | 5.0 | 1.24 | 0.27 |
| TNFa | 72 | 5.0 | 1.26 | 0.27 |
| TNFa | 144 | 4.7 | 1.34 | 0.29 |
