# Supplementary material for: Effect of longevity genotype on acute phase response to a lipopolysaccharide challenge in dairy goats
Source: PLoS One. 2026 Aug 28;21(8):e0356779. doi: 10.1371/journal.pone.0356779 (PMC13524232; doi:10.1371/journal.pone.0356779)
Supplement: Supplementary file 5 [file pone.0356779.s005.docx]

**Supp table 5.** Mean log-transformed concentrations (ln pg/mL) of the eight cytokines not responsive to LPS injection (CXCL10, CXCL8, IL-17a, IL-1α, IL-1β, IL-36RA, MIP1β, VEGFα) at each time point from hour -72 to hour 144 in 83 Alpine dairy goats. Values are presented as mean, standard deviation (SD), and 95% confidence interval of the mean (CI = 1.96 × SD/√n).

| **Cytokine** | **Hour** | **Mean** | **SD** | **95% CI** |
| --- | --- | --- | --- | --- |
| CXCL10 | -72 | 8.1 | 0.32 | 0.07 |
| CXCL10 | 0 | 8.5 | 0.60 | 0.13 |
| CXCL10 | 2 | 8.4 | 0.33 | 0.07 |
| CXCL10 | 4 | 8.5 | 0.48 | 0.10 |
| CXCL10 | 6 | 8.7 | 0.82 | 0.18 |
| CXCL10 | 8 | 8.3 | 0.76 | 0.16 |
| CXCL10 | 10 | 8.7 | 0.37 | 0.08 |
| CXCL10 | 12 | 8.7 | 0.39 | 0.08 |
| CXCL10 | 24 | 8.5 | 0.37 | 0.08 |
| CXCL10 | 48 | 8.0 | 0.70 | 0.15 |
| CXCL10 | 72 | 7.9 | 0.75 | 0.16 |
| CXCL10 | 144 | 7.7 | 0.95 | 0.21 |
| CXCL8 | -72 | 8.8 | 1.00 | 0.21 |
| CXCL8 | 0 | 9.3 | 0.54 | 0.12 |
| CXCL8 | 2 | 9.3 | 0.51 | 0.11 |
| CXCL8 | 4 | 9.0 | 0.61 | 0.13 |
| CXCL8 | 6 | 9.1 | 0.62 | 0.13 |
| CXCL8 | 8 | 8.9 | 0.62 | 0.13 |
| CXCL8 | 10 | 8.8 | 0.67 | 0.15 |
| CXCL8 | 12 | 8.8 | 0.71 | 0.15 |
| CXCL8 | 24 | 8.9 | 0.59 | 0.13 |
| CXCL8 | 48 | 9.1 | 0.38 | 0.08 |
| CXCL8 | 72 | 9.2 | 0.44 | 0.09 |
| CXCL8 | 144 | 9.0 | 0.58 | 0.13 |
| IL17a | -72 | 2.7 | 0.48 | 0.10 |
| IL17a | 0 | 2.7 | 0.54 | 0.12 |
| IL17a | 2 | 2.9 | 0.56 | 0.12 |
| IL17a | 4 | 2.7 | 0.51 | 0.11 |
| IL17a | 6 | 2.6 | 0.50 | 0.11 |
| IL17a | 8 | 2.6 | 0.46 | 0.10 |
| IL17a | 10 | 2.6 | 0.51 | 0.11 |
| IL17a | 12 | 2.6 | 0.50 | 0.11 |
| IL17a | 24 | 2.6 | 0.54 | 0.12 |
| IL17a | 48 | 2.7 | 0.46 | 0.10 |
| IL17a | 72 | 2.7 | 0.49 | 0.11 |
| IL17a | 144 | 2.7 | 0.52 | 0.11 |
| IL1a | -72 | 5.0 | 0.82 | 0.18 |
| IL1a | 0 | 5.3 | 1.09 | 0.23 |
| IL1a | 2 | 5.6 | 1.05 | 0.23 |
| IL1a | 4 | 5.5 | 1.09 | 0.24 |
| IL1a | 6 | 5.3 | 1.09 | 0.23 |
| IL1a | 8 | 5.3 | 1.17 | 0.25 |
| IL1a | 10 | 5.3 | 1.12 | 0.24 |
| IL1a | 12 | 5.3 | 1.07 | 0.23 |
| IL1a | 24 | 5.5 | 1.04 | 0.23 |
| IL1a | 48 | 5.4 | 1.22 | 0.26 |
| IL1a | 72 | 5.5 | 1.16 | 0.25 |
| IL1a | 144 | 5.2 | 1.28 | 0.28 |
| IL1b | -72 | 2.5 | 0.46 | 0.10 |
| IL1b | 0 | 2.6 | 0.42 | 0.09 |
| IL1b | 2 | 2.7 | 0.47 | 0.10 |
| IL1b | 4 | 2.6 | 0.43 | 0.09 |
| IL1b | 6 | 2.7 | 0.33 | 0.07 |
| IL1b | 8 | 2.5 | 0.39 | 0.08 |
| IL1b | 10 | 2.5 | 0.41 | 0.09 |
| IL1b | 12 | 2.5 | 0.43 | 0.09 |
| IL1b | 24 | 2.5 | 0.43 | 0.09 |
| IL1b | 48 | 2.5 | 0.43 | 0.09 |
| IL1b | 72 | 2.5 | 0.41 | 0.09 |
| IL1b | 144 | 2.4 | 0.42 | 0.09 |
| IL36RA | -72 | 6.6 | 0.43 | 0.09 |
| IL36RA | 0 | 7.0 | 0.87 | 0.19 |
| IL36RA | 2 | 6.8 | 0.73 | 0.16 |
| IL36RA | 4 | 6.5 | 0.82 | 0.18 |
| IL36RA | 6 | 6.5 | 0.85 | 0.18 |
| IL36RA | 8 | 6.6 | 0.86 | 0.19 |
| IL36RA | 10 | 6.8 | 0.69 | 0.15 |
| IL36RA | 12 | 7.0 | 0.77 | 0.17 |
| IL36RA | 24 | 6.8 | 0.69 | 0.15 |
| IL36RA | 48 | 6.8 | 0.81 | 0.18 |
| IL36RA | 72 | 6.9 | 0.74 | 0.16 |
| IL36RA | 144 | 6.6 | 1.02 | 0.22 |
| MIP1b | -72 | 3.2 | 0.38 | 0.08 |
| MIP1b | 0 | 3.3 | 0.41 | 0.09 |
| MIP1b | 2 | 3.5 | 0.44 | 0.09 |
| MIP1b | 4 | 3.4 | 0.37 | 0.08 |
| MIP1b | 6 | 3.3 | 0.35 | 0.08 |
| MIP1b | 8 | 3.3 | 0.38 | 0.08 |
| MIP1b | 10 | 3.4 | 0.40 | 0.09 |
| MIP1b | 12 | 3.4 | 0.42 | 0.09 |
| MIP1b | 24 | 3.3 | 0.43 | 0.09 |
| MIP1b | 48 | 3.4 | 0.48 | 0.10 |
| MIP1b | 72 | 3.3 | 0.46 | 0.10 |
| MIP1b | 144 | 3.3 | 0.49 | 0.11 |
| VEGFa | -72 | 6.2 | 0.47 | 0.10 |
| VEGFa | 0 | 6.7 | 0.81 | 0.18 |
| VEGFa | 2 | 6.8 | 0.58 | 0.13 |
| VEGFa | 4 | 6.5 | 0.73 | 0.16 |
| VEGFa | 6 | 6.6 | 0.79 | 0.17 |
| VEGFa | 8 | 6.4 | 0.86 | 0.19 |
| VEGFa | 10 | 6.7 | 0.59 | 0.13 |
| VEGFa | 12 | 6.7 | 0.62 | 0.13 |
| VEGFa | 24 | 6.7 | 0.55 | 0.12 |
| VEGFa | 48 | 6.6 | 0.78 | 0.17 |
| VEGFa | 72 | 6.5 | 0.72 | 0.16 |
| EGFa | 144 | 6.3 | 0.97 | 0.21 |
