# Supplementary material for: Effect of longevity genotype on acute phase response to a lipopolysaccharide challenge in dairy goats
Source: PLoS One. 2026 Aug 28;21(8):e0356779. doi: 10.1371/journal.pone.0356779 (PMC13524232; doi:10.1371/journal.pone.0356779)
Supplement: Supplementary file 6 [file pone.0356779.s006.docx]

**Supp table 6.** Comparison of log-transformed cytokine concentrations (ln pg/mL) between the two longevity lines (High_LGV and Low_LGV) at each time point from hour -72 to hour 144 following LPS injection in 83 Alpine dairy goats. Values are least squares means and 95% confidence intervals estimated from the mixed model [2], correcting for facility effect and including a random animal intercept. P-values correspond to the line contrast at each time point, extracted using the emmeans package. Significance levels: (***) p < 0.001, (**) p < 0.01, (*) p < 0.05, (.) 0.05 < p < 0.10.

| **Cytokine** | **Line** | **Hour** | **Mean** | **95% CI** | **p** | **signif** |
| --- | --- | --- | --- | --- | --- | --- |
| IL6 | High_lGV | -72 | 2.7 | 0.55 | 0.146 |  |
| IL6 | Low_LGV | -72 | 2.9 | 0.56 | 0.146 |  |
| IL6 | High_lGV | 0 | 3.0 | 0.55 | 0.469 |  |
| IL6 | Low_LGV | 0 | 3.2 | 0.56 | 0.469 |  |
| IL6 | High_lGV | 2 | 6.2 | 0.55 | 0.000 | *** |
| IL6 | Low_LGV | 2 | 6.9 | 0.56 | 0.000 | *** |
| IL6 | High_lGV | 4 | 7.6 | 0.55 | 0.011 | * |
| IL6 | Low_LGV | 4 | 8.1 | 0.56 | 0.011 | * |
| IL6 | High_lGV | 6 | 7.9 | 0.55 | 0.052 | . |
| IL6 | Low_LGV | 6 | 8.3 | 0.56 | 0.052 | . |
| IL6 | High_lGV | 8 | 7.4 | 0.55 | 0.029 | * |
| IL6 | Low_LGV | 8 | 7.8 | 0.56 | 0.029 | * |
| IL6 | High_lGV | 10 | 7.3 | 0.55 | 0.038 | * |
| IL6 | Low_LGV | 10 | 7.7 | 0.56 | 0.038 | * |
| IL6 | High_lGV | 12 | 6.8 | 0.55 | 0.005 | ** |
| IL6 | Low_LGV | 12 | 7.3 | 0.56 | 0.005 | ** |
| IL6 | High_lGV | 24 | 5.2 | 0.55 | 0.089 | . |
| IL6 | Low_LGV | 24 | 5.6 | 0.56 | 0.089 | . |
| IL6 | High_lGV | 48 | 3.9 | 0.55 | 0.352 |  |
| IL6 | Low_LGV | 48 | 4.1 | 0.56 | 0.352 |  |
| IL6 | High_lGV | 72 | 3.2 | 0.55 | 0.334 |  |
| IL6 | Low_LGV | 72 | 3.4 | 0.56 | 0.334 |  |
| IL6 | High_lGV | 144 | 3.0 | 0.55 | 0.792 |  |
| IL6 | Low_LGV | 144 | 2.9 | 0.56 | 0.792 |  |
| IL10 | High_lGV | -72 | 5.3 | 0.38 | 0.929 |  |
| IL10 | Low_LGV | -72 | 5.3 | 0.38 | 0.929 |  |
| IL10 | High_lGV | 0 | 5.7 | 0.38 | 0.719 |  |
| IL10 | Low_LGV | 0 | 5.6 | 0.38 | 0.719 |  |
| IL10 | High_lGV | 2 | 7.1 | 0.38 | 0.549 |  |
| IL10 | Low_LGV | 2 | 7.2 | 0.38 | 0.549 |  |
| IL10 | High_lGV | 4 | 7.5 | 0.38 | 0.360 |  |
| IL10 | Low_LGV | 4 | 7.6 | 0.38 | 0.360 |  |
| IL10 | High_lGV | 6 | 7.1 | 0.38 | 0.581 |  |
| IL10 | Low_LGV | 6 | 7.2 | 0.38 | 0.581 |  |
| IL10 | High_lGV | 8 | 6.6 | 0.38 | 0.589 |  |
| IL10 | Low_LGV | 8 | 6.5 | 0.38 | 0.589 |  |
| IL10 | High_lGV | 10 | 6.5 | 0.38 | 0.798 |  |
| IL10 | Low_LGV | 10 | 6.5 | 0.38 | 0.798 |  |
| IL10 | High_lGV | 12 | 6.3 | 0.38 | 0.885 |  |
| IL10 | Low_LGV | 12 | 6.3 | 0.38 | 0.885 |  |
| IL10 | High_lGV | 24 | 5.8 | 0.38 | 0.463 |  |
| IL10 | Low_LGV | 24 | 5.7 | 0.38 | 0.463 |  |
| IL10 | High_lGV | 48 | 5.6 | 0.38 | 0.100 |  |
| IL10 | Low_LGV | 48 | 5.4 | 0.38 | 0.100 |  |
| IL10 | High_lGV | 72 | 5.5 | 0.38 | 0.213 |  |
| IL10 | Low_LGV | 72 | 5.3 | 0.38 | 0.213 |  |
| IL10 | High_lGV | 144 | 5.4 | 0.38 | 0.155 |  |
| IL10 | Low_LGV | 144 | 5.2 | 0.38 | 0.155 |  |
| IFng | High_lGV | -72 | 2.9 | 0.58 | 0.609 |  |
| IFng | Low_LGV | -72 | 3.0 | 0.59 | 0.609 |  |
| IFng | High_lGV | 0 | 2.7 | 0.58 | 0.303 |  |
| IFng | Low_LGV | 0 | 2.9 | 0.59 | 0.303 |  |
| IFng | High_lGV | 2 | 3.2 | 0.58 | 0.146 |  |
| IFng | Low_LGV | 2 | 3.5 | 0.59 | 0.146 |  |
| IFng | High_lGV | 4 | 3.6 | 0.58 | 0.025 | * |
| IFng | Low_LGV | 4 | 4.1 | 0.59 | 0.025 | * |
| IFng | High_lGV | 6 | 3.4 | 0.58 | 0.010 | ** |
| IFng | Low_LGV | 6 | 4.0 | 0.59 | 0.010 | ** |
| IFng | High_lGV | 8 | 3.3 | 0.58 | 0.049 | * |
| IFng | Low_LGV | 8 | 3.7 | 0.59 | 0.049 | * |
| IFng | High_lGV | 10 | 3.2 | 0.58 | 0.088 | . |
| IFng | Low_LGV | 10 | 3.6 | 0.59 | 0.088 | . |
| IFng | High_lGV | 12 | 3.1 | 0.58 | 0.132 |  |
| IFng | Low_LGV | 12 | 3.4 | 0.59 | 0.132 |  |
| IFng | High_lGV | 24 | 2.8 | 0.58 | 0.514 |  |
| IFng | Low_LGV | 24 | 3.0 | 0.59 | 0.514 |  |
| IFng | High_lGV | 48 | 2.8 | 0.58 | 0.952 |  |
| IFng | Low_LGV | 48 | 2.8 | 0.59 | 0.952 |  |
| IFng | High_lGV | 72 | 2.7 | 0.58 | 0.710 |  |
| IFng | Low_LGV | 72 | 2.8 | 0.59 | 0.710 |  |
| IFng | High_lGV | 144 | 2.6 | 0.58 | 0.545 |  |
| IFng | Low_LGV | 144 | 2.7 | 0.59 | 0.545 |  |
| MIP1a | High_lGV | -72 | 5.6 | 0.34 | 0.368 |  |
| MIP1a | Low_LGV | -72 | 5.5 | 0.34 | 0.368 |  |
| MIP1a | High_lGV | 0 | 6.0 | 0.34 | 0.114 |  |
| MIP1a | Low_LGV | 0 | 5.8 | 0.34 | 0.114 |  |
| MIP1a | High_lGV | 2 | 7.0 | 0.34 | 0.928 |  |
| MIP1a | Low_LGV | 2 | 7.0 | 0.34 | 0.928 |  |
| MIP1a | High_lGV | 4 | 6.4 | 0.34 | 0.183 |  |
| MIP1a | Low_LGV | 4 | 6.2 | 0.34 | 0.183 |  |
| MIP1a | High_lGV | 6 | 6.2 | 0.34 | 0.181 |  |
| MIP1a | Low_LGV | 6 | 6.0 | 0.34 | 0.181 |  |
| MIP1a | High_lGV | 8 | 6.1 | 0.34 | 0.021 | * |
| MIP1a | Low_LGV | 8 | 5.8 | 0.34 | 0.021 | * |
| MIP1a | High_lGV | 10 | 6.1 | 0.34 | 0.062 | . |
| MIP1a | Low_LGV | 10 | 5.8 | 0.34 | 0.062 | . |
| MIP1a | High_lGV | 12 | 6.0 | 0.34 | 0.072 | . |
| MIP1a | Low_LGV | 12 | 5.8 | 0.34 | 0.072 | . |
| MIP1a | High_lGV | 24 | 6.0 | 0.34 | 0.055 | . |
| MIP1a | Low_LGV | 24 | 5.8 | 0.34 | 0.055 | . |
| MIP1a | High_lGV | 48 | 6.1 | 0.34 | 0.012 | * |
| MIP1a | Low_LGV | 48 | 5.8 | 0.34 | 0.012 | * |
| MIP1a | High_lGV | 72 | 6.1 | 0.34 | 0.042 | * |
| MIP1a | Low_LGV | 72 | 5.8 | 0.34 | 0.042 | * |
| MIP1a | High_lGV | 144 | 5.8 | 0.34 | 0.036 | * |
| MIP1a | Low_LGV | 144 | 5.6 | 0.34 | 0.036 | * |
| IL4 | High_lGV | -72 | 3.0 | 0.24 | 0.248 |  |
| IL4 | Low_LGV | -72 | 3.1 | 0.24 | 0.248 |  |
| IL4 | High_lGV | 0 | 3.2 | 0.24 | 0.780 |  |
| IL4 | Low_LGV | 0 | 3.3 | 0.24 | 0.780 |  |
| IL4 | High_lGV | 2 | 3.6 | 0.24 | 0.309 |  |
| IL4 | Low_LGV | 2 | 3.5 | 0.24 | 0.309 |  |
| IL4 | High_lGV | 4 | 3.5 | 0.24 | 0.472 |  |
| IL4 | Low_LGV | 4 | 3.5 | 0.24 | 0.472 |  |
| IL4 | High_lGV | 6 | 3.1 | 0.24 | 0.380 |  |
| IL4 | Low_LGV | 6 | 3.2 | 0.24 | 0.380 |  |
| IL4 | High_lGV | 8 | 3.4 | 0.24 | 0.331 |  |
| IL4 | Low_LGV | 8 | 3.3 | 0.24 | 0.331 |  |
| IL4 | High_lGV | 10 | 3.5 | 0.24 | 0.439 |  |
| IL4 | Low_LGV | 10 | 3.4 | 0.24 | 0.439 |  |
| IL4 | High_lGV | 12 | 3.5 | 0.24 | 0.778 |  |
| IL4 | Low_LGV | 12 | 3.5 | 0.24 | 0.778 |  |
| IL4 | High_lGV | 24 | 3.5 | 0.24 | 0.357 |  |
| IL4 | Low_LGV | 24 | 3.4 | 0.25 | 0.357 |  |
| IL4 | High_lGV | 48 | 3.5 | 0.24 | 0.117 |  |
| IL4 | Low_LGV | 48 | 3.4 | 0.25 | 0.117 |  |
| IL4 | High_lGV | 72 | 3.4 | 0.24 | 0.136 |  |
| IL4 | Low_LGV | 72 | 3.3 | 0.25 | 0.136 |  |
| IL4 | High_lGV | 144 | 3.4 | 0.24 | 0.111 |  |
| IL4 | Low_LGV | 144 | 3.2 | 0.25 | 0.111 |  |
| TNFa | High_lGV | -72 | 4.6 | 0.53 | 0.592 |  |
| TNFa | Low_LGV | -72 | 4.5 | 0.53 | 0.592 |  |
| TNFa | High_lGV | 0 | 5.2 | 0.53 | 0.316 |  |
| TNFa | Low_LGV | 0 | 5.0 | 0.53 | 0.316 |  |
| TNFa | High_lGV | 2 | 6.1 | 0.53 | 0.526 |  |
| TNFa | Low_LGV | 2 | 6.2 | 0.53 | 0.526 |  |
| TNFa | High_lGV | 4 | 5.5 | 0.53 | 0.605 |  |
| TNFa | Low_LGV | 4 | 5.4 | 0.53 | 0.605 |  |
| TNFa | High_lGV | 6 | 5.3 | 0.53 | 0.489 |  |
| TNFa | Low_LGV | 6 | 5.1 | 0.53 | 0.489 |  |
| TNFa | High_lGV | 8 | 5.1 | 0.53 | 0.264 |  |
| TNFa | Low_LGV | 8 | 4.9 | 0.53 | 0.264 |  |
| TNFa | High_lGV | 10 | 5.2 | 0.53 | 0.212 |  |
| TNFa | Low_LGV | 10 | 5.0 | 0.53 | 0.212 |  |
| TNFa | High_lGV | 12 | 5.1 | 0.53 | 0.291 |  |
| TNFa | Low_LGV | 12 | 4.9 | 0.53 | 0.291 |  |
| TNFa | High_lGV | 24 | 5.3 | 0.53 | 0.172 |  |
| TNFa | Low_LGV | 24 | 5.0 | 0.53 | 0.172 |  |
| TNFa | High_lGV | 48 | 5.2 | 0.53 | 0.147 |  |
| TNFa | Low_LGV | 48 | 4.9 | 0.53 | 0.147 |  |
| TNFa | High_lGV | 72 | 5.1 | 0.53 | 0.177 |  |
| TNFa | Low_LGV | 72 | 4.8 | 0.53 | 0.177 |  |
| TNFa | High_lGV | 144 | 4.8 | 0.53 | 0.321 |  |
| TNFa | Low_LGV | 144 | 4.6 | 0.53 | 0.321 |  |
